# Radiomics for Predicting Response of Neoadjuvant Chemotherapy in Nasopharyngeal Carcinoma: A Systematic Review and Meta-Analysis

**DOI:** 10.3389/fonc.2022.893103

**Published:** 2022-05-04

**Authors:** Chao Yang, Zekun Jiang, Tingting Cheng, Rongrong Zhou, Guangcan Wang, Di Jing, Linlin Bo, Pu Huang, Jianbo Wang, Daizhou Zhang, Jianwei Jiang, Xing Wang, Hua Lu, Zijian Zhang, Dengwang Li

**Affiliations:** ^1^ Shandong Key Laboratory of Medical Physics and Image Processing, Shandong Institute of Industrial Technology for Health Sciences and Precision Medicine, School of Physics and Electronics, Shandong Normal University, Jinan, China; ^2^ Department of General Practice, Xiangya Hospital, Central South University, Changsha, China; ^3^ National Clinical Research Center for Geriatric Disorders, Xiangya Hospital, Central South University, Changsha, China; ^4^ Department of Oncology, Xiangya Hospital, Central South University, Changsha, China; ^5^ Department of Radiation Oncology, Qilu Hospital, Cheeloo College of Medicine, Shandong University, Jinan, China; ^6^ Shandong Provincial Key Laboratory of Mucosal and Transdermal Drug Delivery Technologies, Shandong Academy of Pharmaceutical Sciences, Jinan, China; ^7^ Optical and Digital Image Processing Division, Qingdao NovelBeam Technology Co., Ltd., Qingdao, China; ^8^ Software Research and Development Center, Shangdong AccurDx Diagnosis of Biotech Co., Ltd., Jinan, China

**Keywords:** nasopharyngeal carcinoma, neoadjuvant chemotherapy, systematic review, meta-analysis, machine learning

## Abstract

**Purpose:**

This study examined the methodological quality of radiomics to predict the effectiveness of neoadjuvant chemotherapy in nasopharyngeal carcinoma (NPC). We performed a meta-analysis of radiomics studies evaluating the bias risk and treatment response estimation.

**Methods:**

Our study was conducted through a literature review as per the Preferred Reporting Items for Systematic Reviews and Meta-Analyses guidelines. We included radiomics-related papers, published prior to January 31, 2022, in our analysis to examine the effectiveness of neoadjuvant chemotherapy in NPC. The methodological quality was assessed using the radiomics quality score. The intra-class correlation coefficient (ICC) was employed to evaluate inter-reader reproducibility. The pooled area under the curve (AUC), pooled sensitivity, and pooled specificity were used to assess the ability of radiomics to predict response to neoadjuvant chemotherapy in NPC. Lastly, the Quality Assessment of Diagnostic Accuracy Studies technique was used to analyze the bias risk.

**Results:**

A total of 12 studies were eligible for our systematic review, and 6 papers were included in our meta-analysis. The radiomics quality score was set from 7 to 21 (maximum score: 36). There was satisfactory ICC (ICC = 0.987, 95% CI: 0.957–0.996). The pooled sensitivity and specificity were 0.88 (95% CI: 0.71–0.95) and 0.82 (95% CI: 0.68–0.91), respectively. The overall AUC was 0.91 (95% CI: 0.88–0.93).

**Conclusion:**

Prediction response of neoadjuvant chemotherapy in NPC using machine learning and radiomics is beneficial in improving standardization and methodological quality before applying it to clinical practice.

## Introduction

Nasopharyngeal carcinoma (NPC) is a malignant head and neck cancer that occurs in the nasopharyngeal space and can spread to the base of the skull and other organs (1–3). Its anatomical location is relatively hidden, causing nearly 70% of NPC patients to be diagnosed at a locally advanced stage (4–6). The pathological subtypes of nasopharyngeal tumors mostly include poorly differentiated or undifferentiated squamous cell carcinomas, which are more sensitive to chemoradiotherapy (7–9). Therefore, definitive concurrent chemoradiotherapy has become the standard of care for NPC patients with locally advanced diseases (10, 11). However, the efficacy of neoadjuvant chemotherapy has been shown to vary greatly in clinical practice, and approximately 30% of patients will develop chemoradiotherapy-related adverse events (12–15).

Radiomics is a highly efficient extraction feature that obtains massive amounts of data from medical images. It transforms imaging data into a high-resolution mineable data space using automated or semiautomated analysis methods (16–18). Given its precise and systematic nature, radiomics can retrieve data that enable the detection of minimal lesions and the prediction of treatment outcomes (19–24). As a result, radiomics is widely used in the study of NPC, and there is huge interest in employing radiomics to predict neoadjuvant chemotherapy efficacy in NPC patients. This information can assist physicians in selecting an optimal scheme and in achieving the maximal anticancer effect. Nevertheless, radiologic data analysis is highly reliant on the subjective interpretation of skilled radiologists. The quantitative data and autonomous imaging markers can serve as an adjunct to expert clinical opinion, thus increasing the prognostic precision (25, 26).

The purpose of this research was to evaluate the methodological quality and analyze the effectiveness of neoadjuvant chemotherapy in NPC among the published radiomics papers. We also performed a meta-analysis of relevant studies to predict the treatment response of neoadjuvant chemotherapy, using the radiomics method, in NPC.

## Materials and Methods

### Study Protocol and Literature Search

This study was conducted as per the Preferred Reporting Items for Systematic Reviews and Meta-Analyses for Diagnostic Test Accuracy (PRISMA-DTA) guidelines (27). Four databases (Web of Science, PubMed, Embase, and Cochrane Library) were screened to select relevant articles published prior to January 31, 2022. The search terms included were as follows: (Nasopharyngeal Carcinoma OR Carcinomas, Nasopharyngeal) AND (Machine Learning OR Artificial Intelligence OR radiomics) AND (CT OR MRI OR Magnetic Resonance Imaging). Please refer to the **Supplementary Material** for more details on the medical subject heading (MESH terms).

Two independent researchers screened article titles and abstracts to determine inclusion in this study. Case reports, non-original publications, and research on topics of interest other than the effectiveness of neoadjuvant chemotherapy in NPC were excluded. To further evaluate relevant articles, the full texts of articles were retrieved and read to determine eligibility for analysis. The reference list of included papers was also reviewed for potential eligible inclusion. The types of images included in our study included MRI, CT, and PET.

### Data Collection

The main endpoints were extracted and adjusted to the largest area under the curve (AUC) in the verification dataset and also prioritized external validation datasets. Among the articles with no external verification dataset, the internal verification dataset (i.e., the test set) was employed. In the absence of an internal verification dataset, the validation set from the training dataset (e.g., leave-one-cross-validation, fivefold cross-validation, and tenfold cross-validation) was employed. The collected models contained radiologically relevant characteristics and sometimes contained characteristics, such as clinical information, pathological types, radiotherapy dose, region of interest (ROI), and imaging features extracted.

### Study Evaluation

The radiomics quality score (RQS) assessed the methodological quality of eligible publications, and the Quality Assessment of Diagnostic Accuracy Studies (QUADAS-2) determined the bias risk (28–30).

RQS assesses an investigation’s methodological quality by examining protocols, images and segmentation reproducibility, feature reduction and verification, biological verification, clinical application, and model performance, with enhanced evidence and open science (28). The detailed RQS report is provided in the **Supplementary Material**. Overall, 16 items were included in the RQS, with scores ranging from −8 to 36. The RQS scores were then converted to percentages, whereby −8 to 0 was 0% and 36 was 100% (28). Two experienced physicians independently scored the RQS of eligible articles.

QUADAS-2 evaluates the bias risk in varying domains (“Patient Selection”, “Index Test”, “Reference Standard”, and “Flow and Timing”) and can be customized to a particular study question. The bias risk for each included study was determined by the QUADAS item of Review Manager 5.4 in order to determine the quality of diagnostic articles (31).

### Meta-Analysis

A meta-analysis of investigations related to the prediction of the treatment response of neoadjuvant chemotherapy in NPC patients was further performed. The data were retrieved by 2 independent reviewers. The internal validity was assessed by a third reviewer. Only studies that provided a two-by-two contingency table or enough data to reconstruct such a table were eligible for analysis. In cases where multiple models were presented, only models with the largest AUC were chosen in our analysis.

### Statistical Analysis

Random-effects meta-analyses were conducted with the restricted maximum likelihood (REML) and presented as a log odds ratio (OR). The threshold effect was determined by calculating the sensitivity and specificity of Spearman’s correlation coefficients. Forest plots and summary receiver operating characteristic (SROC) curve were generated. The pooled AUC, sensitivity, and specificity were used to assess the ability of radiomics to predict the treatment response of neoadjuvant chemotherapy in NPC patients. A funnel plot assessed publication bias. Cochran’s Q test and I^2^ score evaluated heterogeneity among eligible studies. An I^2^ value of 0%–25% meant unremarkable heterogeneity, 25%–50% meant reduced heterogeneity, 50%–75% meant moderate heterogeneity, and >75% meant high heterogeneity (32).

R (version 4.1.2, https://cran.r-project.org/), IBM SPSS Statistics (version 24; IBM Corporation, Armonk, NY, USA), Stata (version 16.0, https://www.stata.com/), and Review Manager (version 5.4) were employed for statistical analyses.

## Results

### Literature Search

**Figure 1:**
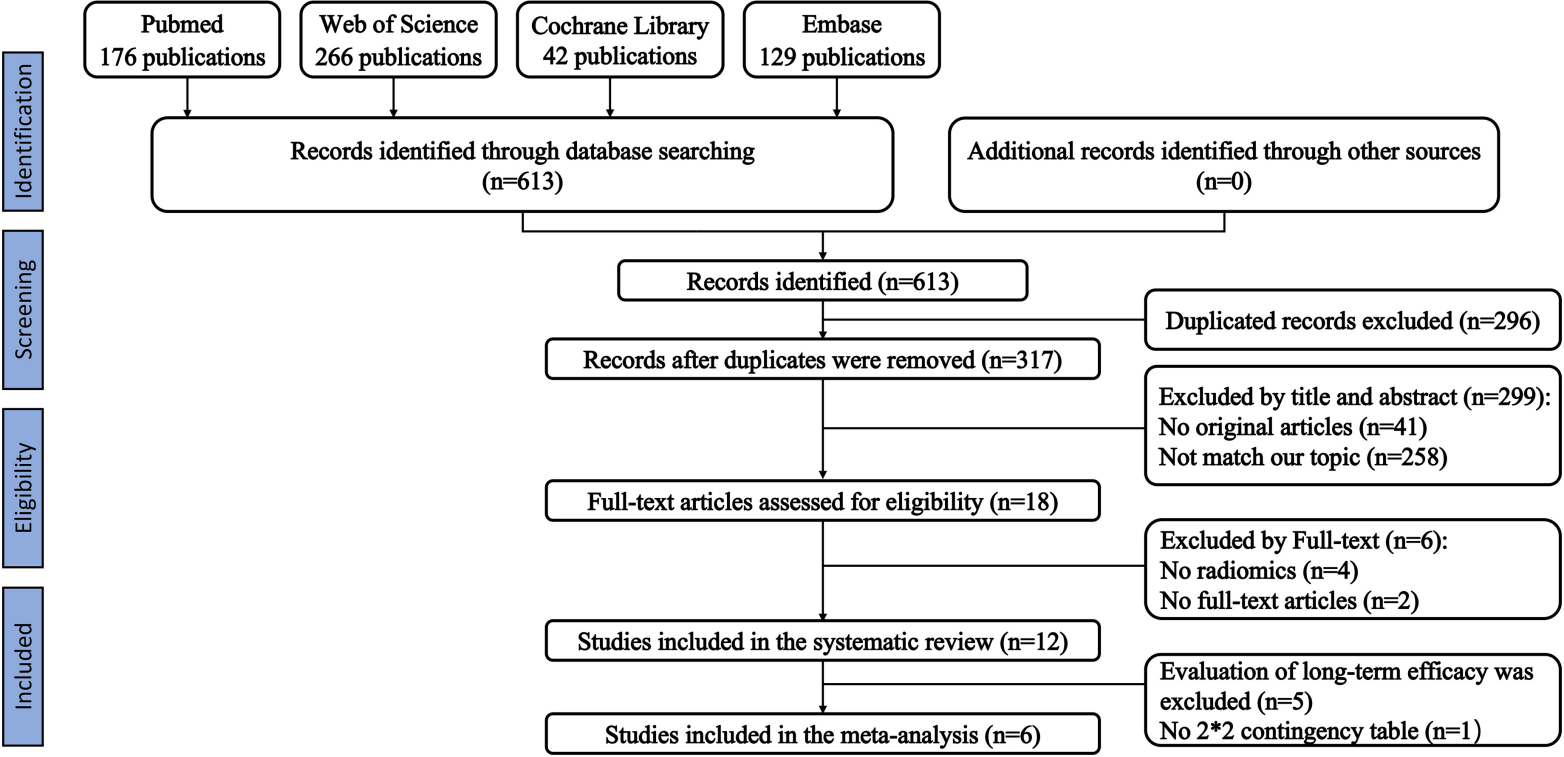
A schematic of the publication selection process.

We initially identified 613 relevant articles; 317 articles were considered for careful evaluation after the elimination of duplicate publications. Upon screening of the titles and abstracts, 18 relevant articles were extracted for further analysis. Four articles that did not contain a radiomics-based model and two conference abstracts were excluded from the analysis. A total of twelve articles that used radiomics-based prediction models were selected for the final systematic review (33–44). Five of the articles examined survival analysis, and seven articles examined the prediction of treatment response. One article that predicted treatment response did not provide enough information to reconstruct a contingency table and calculate the overall outcome (43). Therefore, six articles were included in our meta-analysis. Our PRISMA flowchart is presented in **Figure 1**. The detailed information on all eligible publications is provided in **Table 1**. We summarized detailed information about the selected articles, such as institution, study duration, and type of radiomics features used. The detailed summary table is available in **Tables S4** and **S5**.

**Table 1:** Details of eligible studies.

Author nation, year	Study Type	Cancer	ROI	Imaging	Training set	Test set	External Validation
**Piao China, 2021** (33)	Retrospective observational	NPC	GTVnx	MRI	108	0	0
**Wang China, 2018** (34)	Retrospective observational	NPC	GTVnx	MRI	120	0	0
**Zhang China, 2020** (35)	Retrospective observational	NPC	GTVnx	MRI	81	34	0
**Zhang China, 2020** (36)	Retrospective observational	NPC	GTVnx	MRI	169	19	45
**Chen China, 2021** (37)	Retrospective observational	NPC	GTVnx GTVnd	MRI	847	400	396
**Zhao China, 2020** (38)	Retrospective observational	NPC	GTVnx	MRI	100	23	0
**Peng China, 2019** (39)	Retrospective observational	NPC	GTVnx GTVnd	PET/CT	470	237	0
**Zhong China, 2020** (40)	Retrospective observational	NPC	GTVnx	MRI	447	191	0
**Dong China, 2019** (41)	Retrospective observational	NPC	GTVnx	MRI	254	248	0
**Yang China, 2022** (42)	Retrospective observational	NPC	GTVnx	CT	208	89	0
**Hu China, 2021** (43)	Retrospective observational	NPC	GTVnx GTVnd CTV PTV	MRI	200	84	0
**Liao China, 2021** (44)	Retrospective observational	NPC	GTVnx	MRI	200	86	0

NPC, nasopharyngeal carcinoma; GTVnx, nasopharynx gross tumor volume; GTVnd, lymph node gross tumor volume; CTV, clinical target volume; PTV, planning target volume; MRI, Magnetic Resonance Imaging; CT, Computed Tomography; PET, Positron Emission Tomography.

### Evaluation Criteria for Neoadjuvant Chemotherapy

The response evaluation of neoadjuvant chemotherapy in all included studies was based on the Response Evaluation Criteria in Solid Tumors 1.1 (RECIST 1.1) (45). Complete response (CR) and partial response (PR) were defined as response to treatment, while stable disease (SD) and progressive disease (PD) were defined as no response to treatment.

### Study Evaluation

The RQS scores, ranging from 7 to 21 (maximum score: 36), are summarized in **Table 2**. The publication with the highest RQS percentage was 58.3%. The intra-class correlation coefficient (ICC) between independent reviewers who assessed the publications was 0.987 (95% CI: 0.957–0.996, p < 0.001), which showed excellent reproducibility among readers. The RQS scores examined by the two readers are presented in the **Supplementary Material**. Elevated intra-class association represented the high reliability of quality assessment. Lastly, reviewers reassessed any disagreements until a consensus was reached.

**Table 2:** RQS elements, as reported by Lambin et al. (28), and the mean rating of our eligible studies.

RQS scoring item	Interpretation	Average
Image protocol quality	+1 for well-documented protocols, +1 for publicly available protocols	1.25
Multiple segmentations	+1 if segmented multiple times (different physicians, algorithms, or perturbation of regions of interest)	0.92
Phantom study on all scanners	+1 if texture phantoms were used for feature robustness assessment	0
Imaging at multiple time points	+1 if multiple time points for feature robustness assessment	0
Feature reduction or adjustment for multiple testing	−3 if nothing, +3 if either feature reduction or correction for multiple testing	3
Multivariable analysis with non-radiomics feature	+1 if multivariable analysis with non-radiomics features	0.67
Detect and discuss biological correlates	+1 if present	0.33
Cutoff analyses	+1 if cutoff either predefined or at median or continuous risk variable reported	0.71
Discrimination statistics	+1 for discrimination statistic and statistical significance, +1 if resampling applied	1.75
Calibration statistic	+1 for calibration statistic and statistical significance, +1 if resampling applied	1.17
Prospective study registered in a trial database	+7 for prospective validation within a registered study	0
Validation	−5 if validation is missing, +2 if validation is based on a dataset from the same institute, +3 if validation is based on a dataset from another institute, +4 if validation is based on two datasets from two distinct institutes, +4 if the study validates a previously published signature, +5 if validation is based on three or more datasets from distinct institutes	1.83
Comparison to “gold standard”	+2 for comparison to gold standard	1.83
Potential clinical utility	+2 for reporting potential clinical utility	1.5
Cost-effectiveness analysis	+1 for cost-effectiveness analysis	0
Open science and data	+1 if scans are open source, +1 if region of interest segmentations are open source, +1 if code is open source, +1 if radiomics features are calculated on a set of representative ROIs and the calculated features and representative ROIs are open sources	2.04
Total score (maximum score: 36 points)		17

**Figure 2:**
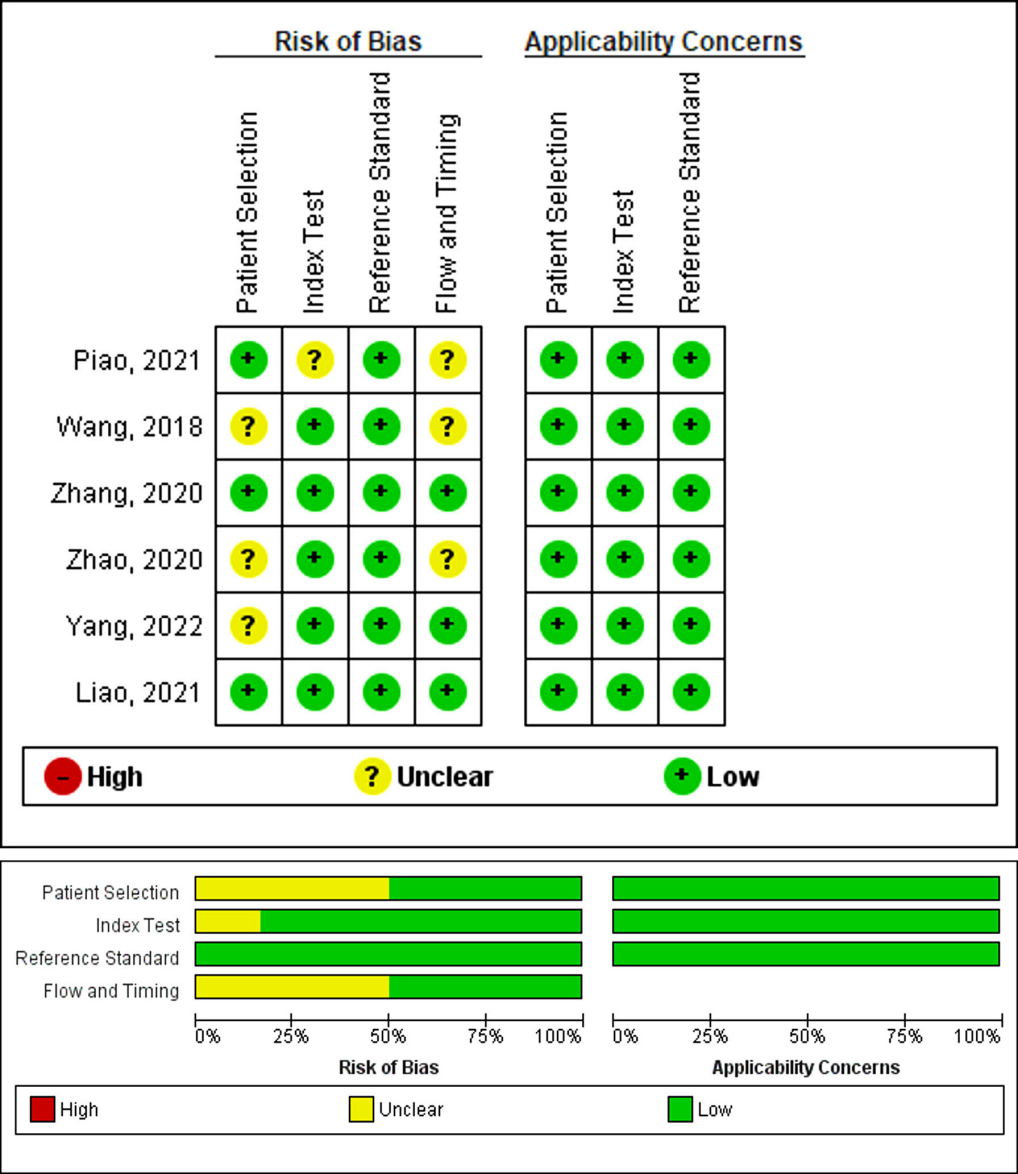
Assessment of the methodological quality of publications included in the meta-analysis, based on the bias risk and applicability using the QUADAS-2 tool. Green, yellow, and red circles denote low, unclear, and high bias risks, respectively.

The bias risk, as assessed by QUADAS-2, is presented in **Figure 2**. The publications with high, unclear, or low bias risk in the four domains of patient selection, index test, reference standard, and flow and timing were 0, 4, and 2, respectively. Particularly, three publications failed to present a clear report of the patient selection process. Therefore, they received an unclear bias risk in the patient selection domain (34, 38, 42). One study received an unclear bias risk in the index test domain (33). Three studies received an unclear bias risk in the flow and timing domain (33, 34, 38). All studies in the meta-analysis displayed relatively reduced concerns regarding applicability in the three domains (patient selection, index test, and reference standard).

### Meta-Analysis

Seven, out of twelve, selected systematic studies discussed the use of radiomics in predicting the treatment response of neoadjuvant chemotherapy. Only six studies provided sufficient data to allow the reconstruction of a contingency table to compute an overall outcome. Hence, only six studies were included in the meta-analysis.

**Figure 3:**
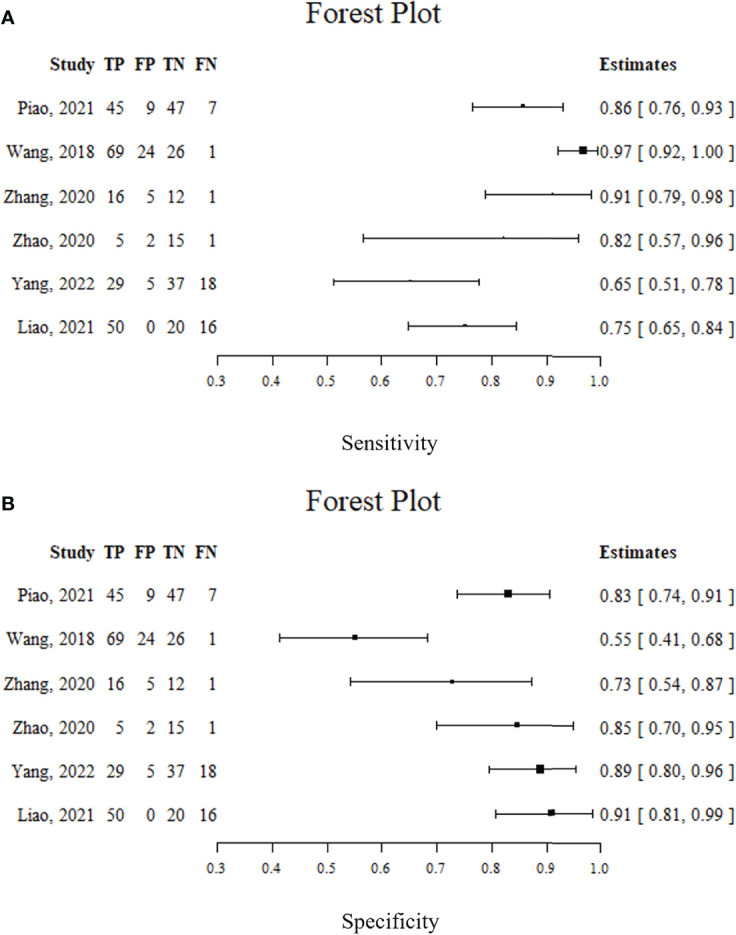
Forest plots. **(A)** sensitivity; **(B)** specificity.

**Figure 4:**
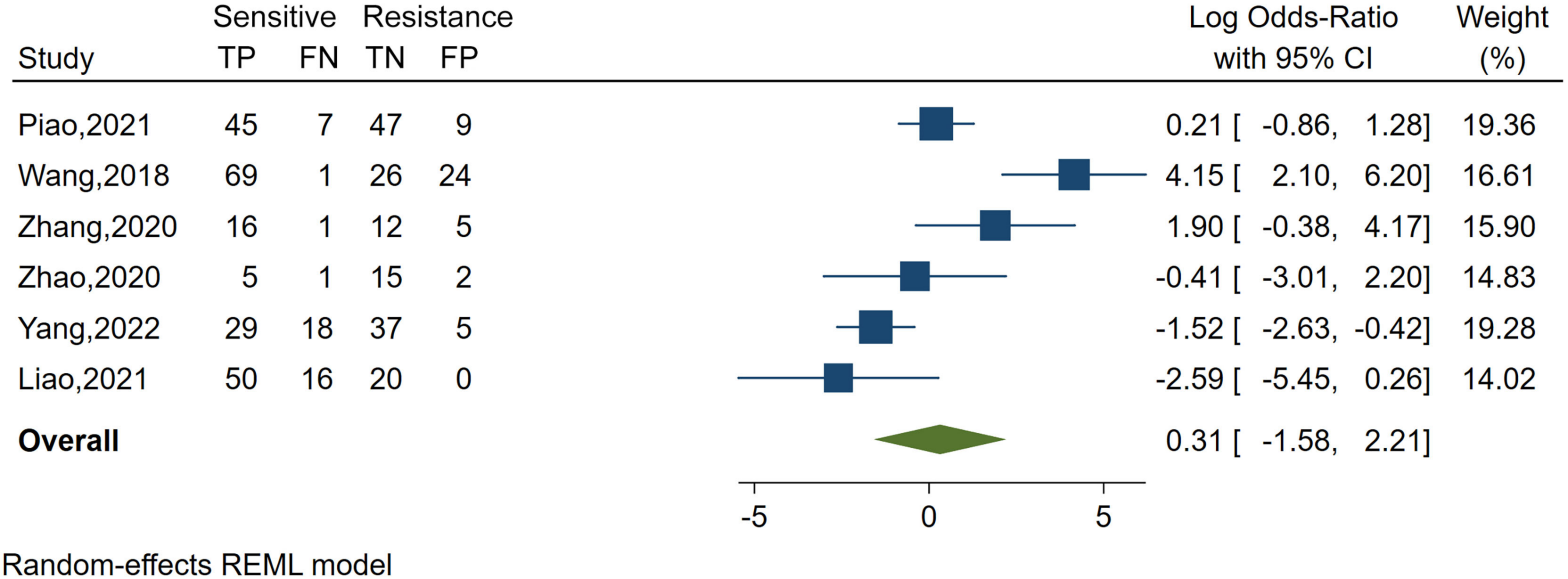
Forest plot of the study outcome, as evidenced by the log odds ratio of six included meta-analysis studies examining the radiomics accuracy in predicting the treatment response to neoadjuvant chemotherapy in treating nasopharyngeal carcinoma. TP, number of patients correctly predicted in the sensitive group; FN, number of patients incorrectly predicted in the resistance group; FP, number of patients incorrectly predicted in the sensitive group; TN, number of patients correctly predicted in the resistance group; x-axis, log-transformed odds ratios; REML, restricted maximum likelihood.

**Figure 5:**
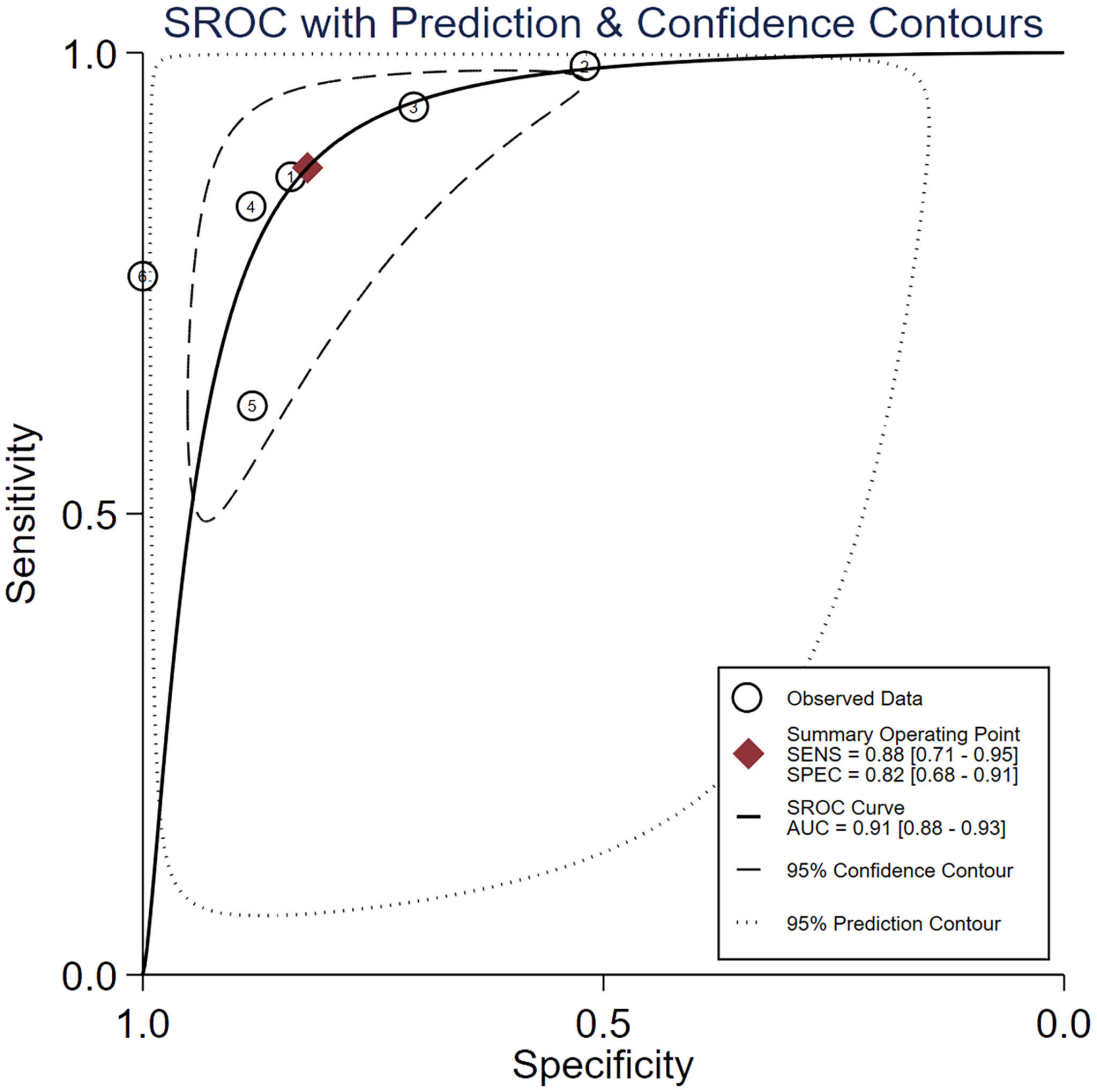
The summary receiver operating characteristic (SROC) curve.

**Figure 6:**
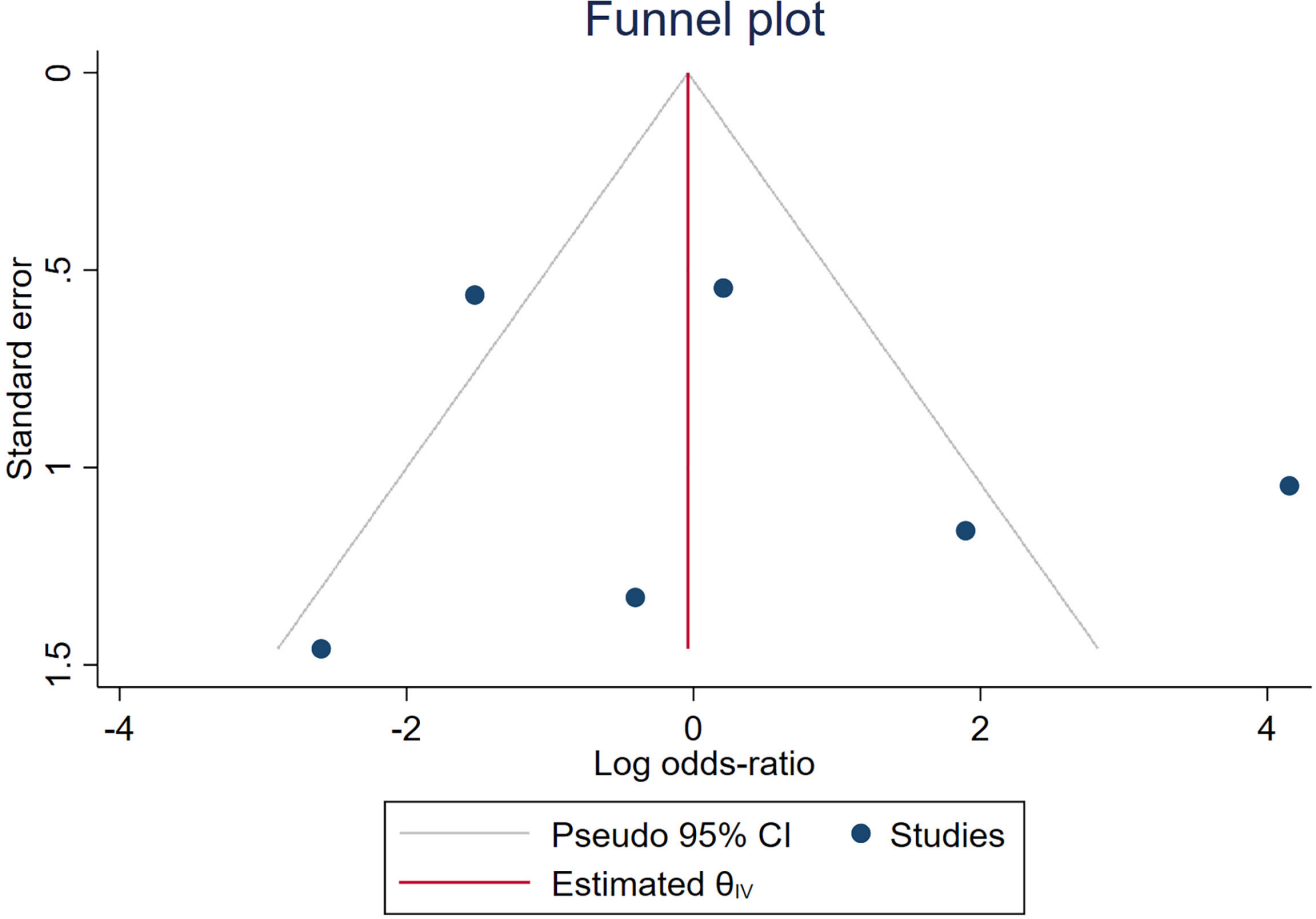
A funnel plot of meta-analyzed studies.

Spearman’s correlation analysis revealed no threshold effect (ρ = 0.486, p = 0.3556). The SROC curve, pooled AUC, pooled sensitivity, and pooled specificity were used to assess the ability of radiomics to predict the response of neoadjuvant chemotherapy in NPC patients. Based on our data analysis, the pooled sensitivity and specificity were 0.88 (95% CI: 0.71–0.95) and 0.82 (95% CI: 0.68–0.91), respectively, as evidenced by the corresponding forest plots in **Figure 3**. The pooled AUC was 0.91 (95% CI: 0.88–0.93). Cochran’s Q was 29.16 (p < 0.01), and the I^2^ score was 85.8%, which represented a high level of heterogeneity within eligible studies with statistically significant heterogeneity. **Figure 4** depicts the forest plot of the treatment outcome, computed as log OR. The log OR of the radiomics model predicting the neoadjuvant chemotherapy treatment response in NPC patients was 0.31 (95% CI: -1.58–2.21). The SROC curve is provided in **Figure 5**. The funnel plot correlating the outcome to standard error is presented in **Figure 6**. Given that we had less than 10 eligible articles in our meta-analysis, Egger’s test was not applicable, as suggested by the Cochrane guidelines (46).

## Discussion

Radiomics has excellent prospects in multiple applications and can potentially aid in retrieving more quantitative data from standard medical images (47). In recent years, radiomics has developed rapidly in NPC research. However, despite ongoing efforts to standardize radiomics extraction features and analysis, their usage outside research is not yet justified (48).

We found several articles based on radiomics to predict response to neoadjuvant chemotherapy in NPC patients, mostly over the last 3 years. This suggested that the use of radiomics in neoadjuvant chemotherapy for NPC patients is novel and remains groundbreaking. Based on our analysis, the characteristics of radiomics investigations were similar among all eligible publications. First, the ROI was manually segmented by two radiologists. Second, the radiomics features were extracted, and relevant features were selected. Third, a model predicting neoadjuvant chemotherapy effectiveness in treating NPC was constructed and evaluated. The texture features were deemed as the most frequent type of radiomics features in the twelve selected articles, and the detailed information is presented in **Table S4**.

Ten of the twelve articles employed texture features in their highest AUC models. The wavelet features were deemed as a frequent occurrence, and others included first-order features and shape features. During the prediction of neoadjuvant chemotherapy efficacy in NPC, radiomics features including texture, wavelet, first order, and other features extracted from images by artificial intelligence algorithms were able to show a lot of hidden information. With an increasing number of radiomics studies, several studies also revealed that textural features could provide additional predictive information (49–53). This systematic analysis found that the Gray Level Run Length Matrix features, the Gray Level Size Zone Matrix features, and the Gray Level Co-occurrence Matrix features are more frequently used. The textural features were shown to provide good results in predicting the efficacy of neoadjuvant chemotherapy treatment for NPC. One possible reason is that texture features contain information related to the efficacy of neoadjuvant chemotherapy treatment.

The advent of radiomics has made great contributions to overcoming limitations of user-dependent interpretation, thus assisting physicians in solving clinical problems. However, it was undeniable that the quality of our current research on radiomics is uneven. RQS is a common method for assessing the quality of radiomics studies and has been shown to accurately evaluate the methodological quality of radiomics studies. This is essential for the critical appraisal of a massive amount of research articles and prioritizing the verification of high-quality data. Since the first RQS application produced certain variations in inter-rater agreements (54), our independent RQS scoring was conducted by two independent readers experienced in radiomics. This way, we achieved a good level of agreement in terms of overall rating (ICC=0.987) and all scoring elements. The ICC, corresponding to each score category, was greater than 0.75. The RQS of our eligible studies were between 7 and 21 points, with a maximum of 36. But all eligible studies were retrospective in nature; therefore, 7 points was lost. We recommend future prospective studies to obtain higher-quality evidence. Moreover, none of the studies we analyzed conducted a cost–benefit analysis, and no phantom investigations were performed in terms of scan images. These deficiencies in research should be resolved in future radiomics research.

Our meta-analysis examined the prediction accuracy of neoadjuvant chemotherapy efficacy in NPC patients, based on radiomics. The SROC curve, obtained from the meta-analysis, is a ROC curve drawn from the OR of different radiomics studies. We demonstrated an enhanced prediction with a pooled AUC of 0.91. Our pooled sensitivity and pooled specificity reached 0.88 and 0.82, respectively. In terms of the publications that were eligible for meta-analysis, our QUADAS-2 assessment revealed a reduced bias risk while highlighting some critical matters. Particularly, three articles exhibited incomplete reporting of the inclusion–exclusion criteria, which can inadvertently introduce bias in the patient selection process (34, 38, 42). Moreover, one study received an unclear bias risk in the index test domain (33), due to the low number of features analyzed to the point of potential bias. In addition, three studies received an unclear bias risk in the flow and timing domain. Among them, one study failed to report the neoadjuvant chemotherapy duration (38). The remaining two studies showed less standardized processes (33, 34), and neither study employed a test set to validate the radiomics model. One study (33) employed a leave-one-out cross-validation method to evaluate the model, and another (34) used the bootstrap-validated model. Although internal validation in the training set can evaluate the performance of the radiomics model, this validation method may have introduced bias. All these concerns are sources of possible bias and should be clearly stated to eliminate bias.

The limitations of our work include the following. First, all studies were retrospective, and no prospective radiomics studies were found. Second, the radiomics features may have been affected by imaging technology. In the future, multicenter prospective investigations should be conducted to fully examine the predictability of radiomics studies (55). Third, RQS is a purely methodological scoring system that does not account for alterations in the study aim. Fourth, our sample size was relatively low, and the included studies were all from China. Fifth, although the QUADAS-2 assessment provided some unclear bias risks, no high bias sources were found. Moreover, being a qualitative score, the QUADAS-2 interpretation is not easily interpretable. Given our small sample size, our publication bias assessment is open to question. Sixth, we noted a high study heterogeneity, but this is typically common among machine learning meta-analyses and diagnostic meta-analyses (56–59).

## Conclusion

Radiomics studies investigating the efficacy of neoadjuvant chemotherapy in NPC patients demonstrated promising results. We, therefore, recommend properly designed prospective trials in the future, including the validation and standardization of methodological data analysis.

## Data Availability Statement

The original contributions presented in the study are included in the article/**Supplementary Material**. Further inquiries can be directed to the corresponding authors.

## Author Contributions

Conceptualization: CY, ZJ, TC, RZ, JW, DZ; Data collection: CY, GW, ZJ, PH; Data analysis: CY, ZJ, ZZ, LB; Data curation: GW, CY, JJ, XW; Writing-original draft preparation: CY; Writing-review and editing: ZJ, DJ, ZZ, HL; Supervision: DL, ZZ and HL. All authors have read and agreed to the published version of the manuscript.

## Funding

This work was funded by the National Natural Science Foundation of China (61971271), the Taishan Scholars Project of Shandong Province (Tsqn20161023), the Jinan City-School Integration Development Strategy Project (JNSX2021023), the Natural Science Foundation of Shandong Province (ZR2019PF011), the Natural Science Foundation of Hunan Province, China (S2021JJKWLH0218), and 2020 Hunan Provincial clinical medical technology innovation guidance project (S2020SFTLJS0217).

## Conflict of Interest

Author DZ is employed by Shandong Provincial Key Laboratory of Transmucosal and Transdermal Drug Delivery, Shandong Freda Pharmaceutical Group Co., Ltd., Shandong Academy of Pharmaceutical Sciences. Author JJ is employed by Qingdao NovelBeam Technology Co., Ltd. Author XW is employed by Shangdong AccurDx Diagnosis of Biotech Co., Ltd. 

The remaining authors declare that the research was conducted in the absence of any commercial or financial relationships that could be construed as a potential conflict of interest.

The handling editor XM declared a shared parent affiliation with the author JW at the time of review.

## Publisher’s Note

All claims expressed in this article are solely those of the authors and do not necessarily represent those of their affiliated organizations, or those of the publisher, the editors and the reviewers. Any product that may be evaluated in this article, or claim that may be made by its manufacturer, is not guaranteed or endorsed by the publisher.

## Abbreviations

AUC, area under curve; CR, complete response; ICC, intra-class correlation coefficient; NPC, nasopharyngeal carcinoma; OR, odds ratio; PD, progressive disease; PR, partial response; PRISMA, Preferred Reporting Items for Systematic Reviews and Meta-Analyses; PRISMA-DTA, Preferred Reporting Items for Systematic Reviews and Meta-Analyses for Diagnostic Test Accuracy; QUADAS-2, Quality Assessment of Diagnostic Accuracy Studies; RECIST, Response Evaluation Criteria in Solid Tumors; REML, restricted maximum likelihood; RQS, radiomics quality score; SD, stable disease; SROC, summary receiver operating characteristic.
