# Supplementary material for: Radiomics for Predicting Response of Neoadjuvant Chemotherapy in Nasopharyngeal Carcinoma: A Systematic Review and Meta-Analysis
Source: Front Oncol. 2022 May 4;12:893103. doi: 10.3389/fonc.2022.893103 (PMC9121398; doi:10.3389/fonc.2022.893103)
Supplement: Supplementary file 1 [file DataSheet_1.docx]

Supplementary Material

**1. Detailed search strategy**

**2. The Radiology Quality Score (RQS)**

**3. The details of the selected articles.**

**1. Detailed search strategy**

**The full string employed for the systematic literature search, formatted for the PubMed search engine.**

(("nasopharyngeal carcinoma"[MeSH Terms] OR ("nasopharyngeal"[All Fields] AND "carcinoma"[All Fields]) OR "nasopharyngeal carcinoma"[All Fields] OR ("nasopharyngeal carcinoma"[MeSH Terms] OR ("nasopharyngeal"[All Fields] AND "carcinoma"[All Fields]) OR "nasopharyngeal carcinoma"[All Fields] OR ("carcinoma"[All Fields] AND "nasopharyngeal"[All Fields]) OR "carcinoma nasopharyngeal"[All Fields]) OR ("nasopharyngeal carcinoma"[MeSH Terms] OR ("nasopharyngeal"[All Fields] AND "carcinoma"[All Fields]) OR "nasopharyngeal carcinoma"[All Fields] OR ("carcinomas"[All Fields] AND "nasopharyngeal"[All Fields]) OR "carcinomas nasopharyngeal"[All Fields]) OR ("nasopharyngeal carcinoma"[MeSH Terms] OR ("nasopharyngeal"[All Fields] AND "carcinoma"[All Fields]) OR "nasopharyngeal carcinoma"[All Fields] OR ("nasopharyngeal"[All Fields] AND "carcinomas"[All Fields]) OR "nasopharyngeal carcinomas"[All Fields]) OR ("nasopharyngeal neoplasms"[MeSH Terms] OR ("nasopharyngeal"[All Fields] AND "neoplasms"[All Fields]) OR "nasopharyngeal neoplasms"[All Fields]) OR ("nasopharyngeal neoplasms"[MeSH Terms] OR ("nasopharyngeal"[All Fields] AND "neoplasms"[All Fields]) OR "nasopharyngeal neoplasms"[All Fields] OR ("nasopharyngeal"[All Fields] AND "neoplasm"[All Fields]) OR "nasopharyngeal neoplasm"[All Fields]) OR ("nasopharyngeal neoplasms"[MeSH Terms] OR ("nasopharyngeal"[All Fields] AND "neoplasms"[All Fields]) OR "nasopharyngeal neoplasms"[All Fields] OR ("neoplasm"[All Fields] AND "nasopharyngeal"[All Fields])) OR ("nasopharyngeal neoplasms"[MeSH Terms] OR ("nasopharyngeal"[All Fields] AND "neoplasms"[All Fields]) OR "nasopharyngeal neoplasms"[All Fields] OR ("neoplasms"[All Fields] AND "nasopharyngeal"[All Fields]) OR "neoplasms nasopharyngeal"[All Fields]) OR ("nasopharyngeal neoplasms"[MeSH Terms] OR ("nasopharyngeal"[All Fields] AND "neoplasms"[All Fields]) OR "nasopharyngeal neoplasms"[All Fields] OR ("nasopharynx"[All Fields] AND "neoplasms"[All Fields]) OR "nasopharynx neoplasms"[All Fields]) OR ("nasopharyngeal neoplasms"[MeSH Terms] OR ("nasopharyngeal"[All Fields] AND "neoplasms"[All Fields]) OR "nasopharyngeal neoplasms"[All Fields] OR ("nasopharynx"[All Fields] AND "neoplasm"[All Fields]) OR "nasopharynx neoplasm"[All Fields]) OR ("nasopharyngeal neoplasms"[MeSH Terms] OR ("nasopharyngeal"[All Fields] AND "neoplasms"[All Fields]) OR "nasopharyngeal neoplasms"[All Fields] OR ("neoplasm"[All Fields] AND "nasopharynx"[All Fields])) OR ("nasopharyngeal neoplasms"[MeSH Terms] OR ("nasopharyngeal"[All Fields] AND "neoplasms"[All Fields]) OR "nasopharyngeal neoplasms"[All Fields] OR ("neoplasms"[All Fields] AND "nasopharynx"[All Fields]) OR "neoplasms nasopharynx"[All Fields]) OR ("nasopharyngeal neoplasms"[MeSH Terms] OR ("nasopharyngeal"[All Fields] AND "neoplasms"[All Fields]) OR "nasopharyngeal neoplasms"[All Fields] OR ("cancer"[All Fields] AND "nasopharynx"[All Fields]) OR "cancer of nasopharynx"[All Fields] OR "nasopharyngeal carcinoma"[MeSH Terms] OR ("nasopharyngeal"[All Fields] AND "carcinoma"[All Fields]) OR "nasopharyngeal carcinoma"[All Fields] OR ("cancer"[All Fields] AND "nasopharynx"[All Fields])) OR ("nasopharyngeal neoplasms"[MeSH Terms] OR ("nasopharyngeal"[All Fields] AND "neoplasms"[All Fields]) OR "nasopharyngeal neoplasms"[All Fields] OR ("nasopharynx"[All Fields] AND "cancers"[All Fields]) OR "nasopharynx cancers"[All Fields]) OR ("nasopharyngeal neoplasms"[MeSH Terms] OR ("nasopharyngeal"[All Fields] AND "neoplasms"[All Fields]) OR "nasopharyngeal neoplasms"[All Fields] OR ("nasopharyngeal"[All Fields] AND "cancer"[All Fields]) OR "nasopharyngeal cancer"[All Fields] OR "nasopharyngeal carcinoma"[MeSH Terms] OR ("nasopharyngeal"[All Fields] AND "carcinoma"[All Fields]) OR "nasopharyngeal carcinoma"[All Fields] OR ("nasopharyngeal"[All Fields] AND "cancer"[All Fields])) OR ("nasopharyngeal neoplasms"[MeSH Terms] OR ("nasopharyngeal"[All Fields] AND "neoplasms"[All Fields]) OR "nasopharyngeal neoplasms"[All Fields] OR ("cancer"[All Fields] AND "nasopharyngeal"[All Fields]) OR "cancer nasopharyngeal"[All Fields]) OR ("nasopharyngeal neoplasms"[MeSH Terms] OR ("nasopharyngeal"[All Fields] AND "neoplasms"[All Fields]) OR "nasopharyngeal neoplasms"[All Fields] OR ("cancers"[All Fields] AND "nasopharyngeal"[All Fields]) OR "cancers nasopharyngeal"[All Fields]) OR ("nasopharyngeal neoplasms"[MeSH Terms] OR ("nasopharyngeal"[All Fields] AND "neoplasms"[All Fields]) OR "nasopharyngeal neoplasms"[All Fields] OR ("nasopharyngeal"[All Fields] AND "cancers"[All Fields]) OR "nasopharyngeal cancers"[All Fields]) OR ("nasopharyngeal neoplasms"[MeSH Terms] OR ("nasopharyngeal"[All Fields] AND "neoplasms"[All Fields]) OR "nasopharyngeal neoplasms"[All Fields] OR ("nasopharynx"[All Fields] AND "cancer"[All Fields]) OR "nasopharynx cancer"[All Fields]) OR ("nasopharyngeal neoplasms"[MeSH Terms] OR ("nasopharyngeal"[All Fields] AND "neoplasms"[All Fields]) OR "nasopharyngeal neoplasms"[All Fields] OR ("cancer"[All Fields] AND "nasopharynx"[All Fields]) OR "cancer nasopharynx"[All Fields]) OR ("nasopharyngeal neoplasms"[MeSH Terms] OR ("nasopharyngeal"[All Fields] AND "neoplasms"[All Fields]) OR "nasopharyngeal neoplasms"[All Fields] OR ("cancers"[All Fields] AND "nasopharynx"[All Fields]) OR "cancers nasopharynx"[All Fields]) OR ("nasopharyngeal neoplasms"[MeSH Terms] OR ("nasopharyngeal"[All Fields] AND "neoplasms"[All Fields]) OR "nasopharyngeal neoplasms"[All Fields] OR ("cancer"[All Fields] AND "nasopharynx"[All Fields]) OR "cancer of the nasopharynx"[All Fields] OR "nasopharyngeal carcinoma"[MeSH Terms] OR ("nasopharyngeal"[All Fields] AND "carcinoma"[All Fields]) OR "nasopharyngeal carcinoma"[All Fields] OR ("cancer"[All Fields] AND "nasopharynx"[All Fields]))) AND ((((((((((((("machine learning"[MeSH Terms] OR ("machine"[All Fields] AND "learning"[All Fields]) OR "machine learning"[All Fields] OR ("machine learning"[MeSH Terms] OR ("machine"[All Fields] AND "learning"[All Fields]) OR "machine learning"[All Fields] OR ("learning"[All Fields] AND "machine"[All Fields]) OR "learning machine"[All Fields]) OR ("machine learning"[MeSH Terms] OR ("machine"[All Fields] AND "learning"[All Fields]) OR "machine learning"[All Fields] OR ("transfer"[All Fields] AND "learning"[All Fields]) OR "transfer learning"[All Fields]) OR ("transfer, psychology"[MeSH Terms] OR ("transfer"[All Fields] AND "psychology"[All Fields]) OR "psychology transfer"[All Fields] OR ("learning"[All Fields] AND "transfer"[All Fields]) OR "learning transfer"[All Fields]) OR ("artificial intelligence"[MeSH Terms] OR ("artificial"[All Fields] AND "intelligence"[All Fields]) OR "artificial intelligence"[All Fields]) OR ("artificial intelligence"[MeSH Terms] OR ("artificial"[All Fields] AND "intelligence"[All Fields]) OR "artificial intelligence"[All Fields] OR ("intelligence"[All Fields] AND "artificial"[All Fields]) OR "intelligence artificial"[All Fields]) OR ("artificial intelligence"[MeSH Terms] OR ("artificial"[All Fields] AND "intelligence"[All Fields]) OR "artificial intelligence"[All Fields] OR ("computational"[All Fields] AND "intelligence"[All Fields]) OR "computational intelligence"[All Fields]) OR ("artificial intelligence"[MeSH Terms] OR ("artificial"[All Fields] AND "intelligence"[All Fields]) OR "artificial intelligence"[All Fields] OR ("intelligence"[All Fields] AND "computational"[All Fields]) OR "intelligence computational"[All Fields]) OR ("artificial intelligence"[MeSH Terms] OR ("artificial"[All Fields] AND "intelligence"[All Fields]) OR "artificial intelligence"[All Fields] OR ("machine"[All Fields] AND "intelligence"[All Fields]) OR "machine intelligence"[All Fields]) OR ("artificial intelligence"[MeSH Terms] OR ("artificial"[All Fields] AND "intelligence"[All Fields]) OR "artificial intelligence"[All Fields] OR ("intelligence"[All Fields] AND "machine"[All Fields]) OR "intelligence machine"[All Fields]) OR ("artificial intelligence"[MeSH Terms] OR ("artificial"[All Fields] AND "intelligence"[All Fields]) OR "artificial intelligence"[All Fields] OR ("computer"[All Fields] AND "reasoning"[All Fields]) OR "computer reasoning"[All Fields]) OR ("artificial intelligence"[MeSH Terms] OR ("artificial"[All Fields] AND "intelligence"[All Fields]) OR "artificial intelligence"[All Fields] OR ("reasoning"[All Fields] AND "computer"[All Fields]) OR "reasoning computer"[All Fields]) OR ("antagonists and inhibitors"[MeSH Subheading] OR ("antagonists"[All Fields] AND "inhibitors"[All Fields]) OR "antagonists and inhibitors"[All Fields] OR "ai"[All Fields])) AND ("artificial intelligence"[MeSH Terms] OR ("artificial"[All Fields] AND "intelligence"[All Fields]) OR "artificial intelligence"[All Fields])) OR ("artificial intelligence"[MeSH Terms] OR ("artificial"[All Fields] AND "intelligence"[All Fields]) OR "artificial intelligence"[All Fields] OR ("computer"[All Fields] AND "vision"[All Fields] AND "systems"[All Fields]) OR "computer vision systems"[All Fields]) OR ("artificial intelligence"[MeSH Terms] OR ("artificial"[All Fields] AND "intelligence"[All Fields]) OR "artificial intelligence"[All Fields] OR ("computer"[All Fields] AND "vision"[All Fields] AND "system"[All Fields]) OR "computer vision system"[All Fields]) OR ("artificial intelligence"[MeSH Terms] OR ("artificial"[All Fields] AND "intelligence"[All Fields]) OR "artificial intelligence"[All Fields] OR ("system"[All Fields] AND "computer"[All Fields] AND "vision"[All Fields]) OR "system computer vision"[All Fields]) OR ("artificial intelligence"[MeSH Terms] OR ("artificial"[All Fields] AND "intelligence"[All Fields]) OR "artificial intelligence"[All Fields] OR ("systems"[All Fields] AND "computer"[All Fields] AND "vision"[All Fields]) OR "systems computer vision"[All Fields]) OR ("artificial intelligence"[MeSH Terms] OR ("artificial"[All Fields] AND "intelligence"[All Fields]) OR "artificial intelligence"[All Fields] OR ("vision"[All Fields] AND "system"[All Fields] AND "computer"[All Fields])) OR ("artificial intelligence"[MeSH Terms] OR ("artificial"[All Fields] AND "intelligence"[All Fields]) OR "artificial intelligence"[All Fields] OR ("vision"[All Fields] AND "systems"[All Fields] AND "computer"[All Fields])) OR ("education"[MeSH Terms] OR "education"[All Fields] OR ("knowledge"[All Fields] AND "acquisition"[All Fields]) OR "knowledge acquisition"[All Fields])) AND ("computability"[All Fields] OR "computable"[All Fields] OR "computating"[All Fields] OR "computation"[All Fields] OR "computational"[All Fields] OR "computations"[All Fields] OR "compute"[All Fields] OR "computed"[All Fields] OR "computer s"[All Fields] OR "computers"[MeSH Terms] OR "computers"[All Fields] OR "computer"[All Fields] OR "computes"[All Fields] OR "computing"[All Fields] OR "computional"[All Fields])) OR (("acquisition"[All Fields] OR "acquisitions"[All Fields]) AND ("knowledge"[MeSH Terms] OR "knowledge"[All Fields] OR "knowledge s"[All Fields] OR "knowledgeability"[All Fields] OR "knowledgeable"[All Fields] OR "knowledgeably"[All Fields] OR "knowledges"[All Fields]))) AND ("computability"[All Fields] OR "computable"[All Fields] OR "computating"[All Fields] OR "computation"[All Fields] OR "computational"[All Fields] OR "computations"[All Fields] OR "compute"[All Fields] OR "computed"[All Fields] OR "computer s"[All Fields] OR "computers"[MeSH Terms] OR "computers"[All Fields] OR "computer"[All Fields] OR "computes"[All Fields] OR "computing"[All Fields] OR "computional"[All Fields])) OR (("knowledge"[MeSH Terms] OR "knowledge"[All Fields] OR "knowledge s"[All Fields] OR "knowledgeability"[All Fields] OR "knowledgeable"[All Fields] OR "knowledgeably"[All Fields] OR "knowledges"[All Fields]) AND ("representability"[All Fields] OR "representable"[All Fields] OR "representation"[All Fields] OR "representation s"[All Fields] OR "representational"[All Fields] OR "representations"[All Fields]))) AND ("computability"[All Fields] OR "computable"[All Fields] OR "computating"[All Fields] OR "computation"[All Fields] OR "computational"[All Fields] OR "computations"[All Fields] OR "compute"[All Fields] OR "computed"[All Fields] OR "computer s"[All Fields] OR "computers"[MeSH Terms] OR "computers"[All Fields] OR "computer"[All Fields] OR "computes"[All Fields] OR "computing"[All Fields] OR "computional"[All Fields])) OR (("knowledge"[MeSH Terms] OR "knowledge"[All Fields] OR "knowledge s"[All Fields] OR "knowledgeability"[All Fields] OR "knowledgeable"[All Fields] OR "knowledgeably"[All Fields] OR "knowledges"[All Fields]) AND ("representability"[All Fields] OR "representable"[All Fields] OR "representation"[All Fields] OR "representation s"[All Fields] OR "representational"[All Fields] OR "representations"[All Fields]))) AND ("computability"[All Fields] OR "computable"[All Fields] OR "computating"[All Fields] OR "computation"[All Fields] OR "computational"[All Fields] OR "computations"[All Fields] OR "compute"[All Fields] OR "computed"[All Fields] OR "computer s"[All Fields] OR "computers"[MeSH Terms] OR "computers"[All Fields] OR "computer"[All Fields] OR "computes"[All Fields] OR "computing"[All Fields] OR "computional"[All Fields])) OR (("representability"[All Fields] OR "representable"[All Fields] OR "representation"[All Fields] OR "representation s"[All Fields] OR "representational"[All Fields] OR "representations"[All Fields]) AND ("knowledge"[MeSH Terms] OR "knowledge"[All Fields] OR "knowledge s"[All Fields] OR "knowledgeability"[All Fields] OR "knowledgeable"[All Fields] OR "knowledgeably"[All Fields] OR "knowledges"[All Fields]))) AND ("computability"[All Fields] OR "computable"[All Fields] OR "computating"[All Fields] OR "computation"[All Fields] OR "computational"[All Fields] OR "computations"[All Fields] OR "compute"[All Fields] OR "computed"[All Fields] OR "computer s"[All Fields] OR "computers"[MeSH Terms] OR "computers"[All Fields] OR "computer"[All Fields] OR "computes"[All Fields] OR "computing"[All Fields] OR "computional"[All Fields])) OR ("algorithm s"[All Fields] OR "algorithmic"[All Fields] OR "algorithmically"[All Fields] OR "algorithmics"[All Fields] OR "algorithmization"[All Fields] OR "algorithms"[MeSH Terms] OR "algorithms"[All Fields] OR "algorithm"[All Fields]) OR ("algorithm s"[All Fields] OR "algorithmic"[All Fields] OR "algorithmically"[All Fields] OR "algorithmics"[All Fields] OR "algorithmization"[All Fields] OR "algorithms"[MeSH Terms] OR "algorithms"[All Fields] OR "algorithm"[All Fields]) OR ("radiomic"[All Fields] OR "radiomics"[All Fields])) AND ("j comput tomogr"[Journal] OR "commun theory"[Journal] OR "child teenagers"[Journal] OR "cancer ther"[Journal] OR "ct"[All Fields] OR ("magnetic resonance imaging"[MeSH Terms] OR ("magnetic"[All Fields] AND "resonance"[All Fields] AND "imaging"[All Fields]) OR "magnetic resonance imaging"[All Fields] OR "mri"[All Fields]) OR ("magnetic resonance imaging"[MeSH Terms] OR ("magnetic"[All Fields] AND "resonance"[All Fields] AND "imaging"[All Fields]) OR "magnetic resonance imaging"[All Fields]))) AND (2000:2022[pdat]).

**2. The Radiology Quality Score (RQS)**

**Table S1. Details of RQS (S1).**

| **Criteria** | **Points** |
| --- | --- |
| Image protocol quality - well-documented image protocols (for example, contrast, slice thickness, energy, etc.) and/or usage of public image protocols allow reproducibility/replicability | + 1 (if protocols are well-documented) + 1 (if public protocol is used) |
| Multiple segmentations - possible actions are: segmentation by different physicians/algorithms/software, perturbing segmentations by (random) noise, segmentation at different breathing cycles. Analyse feature robustness to segmentation variabilities | + 1 |
| Phantom study on all scanners - detect inter-scanner differences and vendor-dependent features. Analyse feature robustness to these sources of variability | + 1 |
| Imaging at multiple time points - collect images of individuals at additional time points. Analyse feature robustness to temporal variabilities (for example, organ movement, organ expansion/shrinkage) | + 1 |
| Feature reduction or adjustment for multiple testing - decreases the risk of overfitting. Overfitting is inevitable if the number of features exceeds the number of samples. Consider feature robustness when selecting features | - 3 (if neither measure is implemented) + 3 (if either measure is implemented) |
| Multivariable analysis with non radiomics features (for example, EGFR mutation) - is expected to provide a more holistic model. Permits correlating/inferencing between radiomics and non radiomics features | + 1 |
| Detect and discuss biological correlates-demonstration of phenotypic differences (possibly associated with underlying gene–protein expression patterns) deepens understanding of radiomics and biology | + 1 |
| Cut-off analyses - determine risk groups by either the median, a previously published cut-off or report a continuous risk variable. Reduces the risk of reporting overly optimistic results | + 1 |
| Discrimination statistics - report discrimination statistics (for example, C-statistic, ROC curve, AUC) and their statistical significance (for example, p-values, confidence intervals). One can also apply resampling method (for example, bootstrapping, cross-validation) | + 1 (if a discrimination statistic and its statistical significance are reported) + 1 (if a resampling method technique is also applied) |
| Calibration statistics - report calibration statistics (for example, Calibration-in-the-large/slope, calibration plots) and their statistical significance (for example, P-values, confidence intervals). One can also apply resampling method (for example, bootstrapping, cross-validation) | + 1 (if a calibration statistic and its statistical significance are reported) + 1 (if a resampling method technique is also applied) |
| Prospective study registered in a trial database -provides the highest level of evidence supporting the clinical validity and usefulness of the radiomics biomarker | + 7 (for prospective validation of a radiomics signature in an appropriate trial) |
| Validation - the validation is performed without retraining and without adaptation of the cut-off value, provides crucial information with regard to credible clinical performance | - 5 (if validation is missing) + 2 (if validation is based on a dataset from the same institute) + 3 (if validation is based on a dataset from another institute) + 4 (if validation is based on two datasets from two  distinct institutes) + 4 (if the study validates a previously published  signature) + 5 (if validation is based on three or more datasets from distinct institutes)  *Datasets should be of comparable size and should have at least 10 events per model feature |
| Comparison to ‘gold standard’ - assess the extent to which the model agrees with/is superior to the current ‘gold standard’ method (for example, TNM-staging for survival prediction). This comparison shows the added value of radiomics | + 2 |
| Potential clinical utility - report on the current and potential application of the model in a clinical setting (for example, decision curve analysis). | + 2 |
| Cost-effectiveness analysis - report on the cost-effectiveness of the clinical application (for example, QALYs generated) | + 1 |
| Open science and data - make code and data publicly available. Open science facilitates knowledge transfer and reproducibility of the study | + 1 (if scans are open source) + 1 (if region of interest segmentations are open source) + 1 (if code is open source)  + 1 (if radiomics features are calculated on a set of representative ROIs and the calculated features and representative ROIs are open source) |
| Total points (36 = 100%) | |

**Table S2. The RQS for review1**

| Study Criteria | Piao  2021 | Wang  2018 | Zhang  2020 | Zhang  2020 | Chen  2020 | Zhao  2020 | Peng  2019 | Zhong  2020 | Dong  2019 | Yang  2022 | Hu  2021 | Liao  2021 |
| --- | --- | --- | --- | --- | --- | --- | --- | --- | --- | --- | --- | --- |
| Image protocol quality | +2 | +2 | +2 | +2 | +0 | +0 | +2 | +2 | +2 | +2 | +1 | +2 |
| Multiple segmentations | +1 | +0 | +1 | +1 | +1 | +1 | +1 | +1 | +1 | +1 | +1 | +1 |
| Phantom study on all scanners | +0 | +0 | +0 | +0 | +0 | +0 | +0 | +0 | +0 | +0 | +0 | +0 |
| Imaging at multiple time points | +0 | +0 | +0 | +0 | +0 | +0 | +0 | +0 | +0 | +0 | +0 | +0 |
| Feature reduction or adjustment for multiple testing | +3 | +3 | +3 | +3 | +3 | +3 | +3 | +3 | +3 | +3 | +3 | +3 |
| Multivariable analysis with non-radiomics feature | +0 | +0 | +0 | +1 | +1 | +1 | +1 | +1 | +0 | +1 | +1 | +1 |
| Detect and discuss biological correlates | +0 | +0 | +1 | +0 | +0 | +1 | +1 | +0 | +1 | +0 | +0 | +0 |
| Cutoff analyses | +1 | +1 | +1 | +1 | +1 | +1 | +1 | +1 | +1 | +0 | +0 | +0 |
| Discrimination statistics | +1 | +2 | +1 | +2 | +2 | +2 | +2 | +2 | +2 | +2 | +2 | +2 |
| Calibration statistic | +1 | +2 | +2 | +1 | +1 | +1 | +1 | +1 | +1 | +1 | +1 | +1 |
| Prospective study registered in a trial database | +0 | +0 | +0 | +0 | +0 | +0 | +0 | +0 | +0 | +0 | +0 | +0 |
| Validation | -5 | +2 | +2 | +4 | +5 | +2 | +2 | +2 | +2 | +2 | +2 | +2 |
| Comparison to “gold standard” | +2 | +0 | +2 | +2 | +2 | +2 | +2 | +2 | +2 | +2 | +2 | +2 |
| Potential clinical utility | +0 | +0 | +0 | +2 | +2 | +2 | +2 | +2 | +2 | +2 | +2 | +2 |
| Cost-effectiveness analysis | +0 | +0 | +0 | +0 | +0 | +0 | +0 | +0 | +0 | +0 | +0 | +0 |
| Open science and data | +2 | +2 | +2 | +2 | +3 | +1 | +2 | +2 | +2 | +2 | +2 | +2 |
| Total score (Maximum:36) | 8 | 14 | 17 | 21 | 21 | 17 | 20 | 19 | 19 | 18 | 17 | 18 |

**Table S3. The RQS for review2**

| Study Criteria | Piao  2021 | Wang  2018 | Zhang  2020 | Zhang  2020 | Chen  2020 | Zhao  2020 | Peng  2019 | Zhong  2020 | Dong  2019 | Yang  2022 | Hu  2021 | Liao  2021 |
| --- | --- | --- | --- | --- | --- | --- | --- | --- | --- | --- | --- | --- |
| Image protocol quality | +1 | +1 | +1 | +1 | +0 | +0 | +2 | +1 | +1 | +1 | +1 | +1 |
| Multiple segmentations | +1 | +0 | +1 | +1 | +1 | +1 | +1 | +1 | +1 | +1 | +1 | +1 |
| Phantom study on all scanners | +0 | +0 | +0 | +0 | +0 | +0 | +0 | +0 | +0 | +0 | +0 | +0 |
| Imaging at multiple time points | +0 | +0 | +0 | +0 | +0 | +0 | +0 | +0 | +0 | +0 | +0 | +0 |
| Feature reduction or adjustment for multiple testing | +3 | +3 | +3 | +3 | +3 | +3 | +3 | +3 | +3 | +3 | +3 | +3 |
| Multivariable analysis with non-radiomics feature | +0 | +0 | +0 | +1 | +1 | +1 | +1 | +1 | +0 | +1 | +1 | +1 |
| Detect and discuss biological correlates | +0 | +0 | +1 | +0 | +0 | +1 | +1 | +0 | +1 | +0 | +0 | +0 |
| Cutoff analyses | +1 | +1 | +1 | +1 | +1 | +1 | +1 | +0 | +1 | +0 | +0 | +0 |
| Discrimination statistics | +1 | +2 | +1 | +2 | +2 | +2 | +2 | +2 | +2 | +1 | +1 | +2 |
| Calibration statistic | +1 | +2 | +2 | +1 | +1 | +1 | +1 | +1 | +1 | +1 | +1 | +1 |
| Prospective study registered in a trial database | +0 | +0 | +0 | +0 | +0 | +0 | +0 | +0 | +0 | +0 | +0 | +0 |
| Validation | -5 | +2 | +2 | +4 | +5 | +2 | +2 | +2 | +2 | +2 | +2 | +2 |
| Comparison to “gold standard” | +2 | +0 | +2 | +2 | +2 | +2 | +2 | +2 | +2 | +2 | +2 | +2 |
| Potential clinical utility | +0 | +0 | +0 | +2 | +2 | +2 | +2 | +2 | +2 | +2 | +2 | +2 |
| Cost-effectiveness analysis | +0 | +0 | +0 | +0 | +0 | +0 | +0 | +0 | +0 | +0 | +0 | +0 |
| Open science and data | +2 | +2 | +2 | +2 | +3 | +1 | +2 | +2 | +2 | +3 | +2 | +2 |
| Total score (Maximum:36) | 7 | 13 | 16 | 20 | 21 | 17 | 20 | 17 | 18 | 17 | 16 | 17 |

**3. The details of the selected articles.**

**Table S4. The type of features used for the highest AUC model in the validation set or training set.**

| Author Nation, Year | Data source | Time of therapy | Radiomics feature type | Other features |
| --- | --- | --- | --- | --- |
| Piao China, 2021 | Cancer Hospital of the University of Chinese Academy of Sciences | 2016.01-2016.12 | Texture features | - |
| Wang China, 2018 | Guangdong General Hospital | 2009.08-2016.05 | Texture features | - |
| Zhang China, 2020 | Cancer Hospital of the University of Chinese Academy of Sciences | 2018.01-2020.04 | Shape, texture features | - |
| Zhang China, 2020 | The First Affiliated Hospital of Jinan University  Zhongshan Hospital of Sun Yat-sen University | 2009.03-2018.12  2008.08-2016.12 | Shape, wavelet, first-order, texture features | TNM, pEBV-DNA, deep learning features |
| Chen China, 2021 | Sun Yat-sen University Cancer Center  Dongguan People’s Hospital  First People’s Hospital of Foshan | 2011-2016 | shape | TNM, pEBV-DNA, tumor burden variables |
| Zhao China, 2020 | Xijing Hospital | 2012.01-2016.12 | First-order, texture features | Age, gender, TNM, pEBV-DNA |
| Peng China, 2019 | Sun Yat-sen University Cancer Center | 2009.12-2014.12 | Histogram, texture features | Deep learning features |
| Zhong China, 2020 | Sun Yat-sen University Cancer Center | 2010.01-2016.03 | - | Age, gender, pre pEBV-DNA, C-reaction protein, LDH, deep learning features |
| Dong China, 2019 | Sun Yat-sen University Cancer Center | 2011.03-2013.08 | Intensity, texture features | Age, gender, pEBV-DNA, cervical nodal necrosis, primary tumor volume |
| Yang China, 2022 | West China Hospital | 2012.01-2018.12 | First-order, wavelet, texture features | Age, gender, smoking, drinking, AJCC, WHO, pre pEBV-DNA, LDH, deep learning features |
| Hu China,  2021 | Fujian Medical University Cancer Hospital | 2014.01-2015.07 | Shape, wavelet, first-order, texture features | Gender, TNM, AJCC, pEBV-DNA |
| Liao China, 2021 | Guangxi Medical University Cancer Hospital | 2015.01-2018.06 | Wavelet, exponential, texture features | Gender, TNM, pEBV-DNA |

TNM, tumor-node-metastasis; pEBV-DNA, plasma EBV-DNA; LDH, serum level of lactate dehydrogenase; AJCC, American Joint Committee on Cancer overall stage; WHO, WHO pathology subtype.

**Table S5. Overview of the primary endpoint, feature selection methods, feature extraction, predictive models and statistical analyses included in the study.**

| Author Nation, Year | Primary Endpoint | Feature Selection | Feature Extraction | Predictive Model | Statistical Analyses |
| --- | --- | --- | --- | --- | --- |
| Piao China, 2021 | Treatment Response | ANOVA/MW test, correlation analysis, LASSO | AI Kit software | Multivariate logistic regressions | SPSS, R, ROC |
| Wang China, 2018 | Treatment Response | LASSO logistic regression | Matlab | Radiomics signature (Rad score) | SPSS, R, ROC |
| Zhang China, 2020 | Treatment Response | ICC, mRMR, LASSO | AI Kit software | Radiomics signature (Rad score) | R, ROC |
| Zhang China, 2020 | DMFS | ResNet; univariate and multivariate analyses, mRMR, LASSO, AIC | Python DL Pyradiomics | DL signature; Radiomics signature (Rad score) | Python, R, ROC, Kaplan Meier survival curves |
| Chen China, 2021 | DMFS | - | - | XGBoost, LASSO | Python, R, ROC, DCA |
| Zhao China, 2020 | Treatment Response | T test, LASSO | Matlab | SVM, RF | R, ROC, DCA, Kaplan Meier survival curves, nomogram |
| Peng China, 2019 | DFS | LASSO Cox regression, ICC, pearson, univariate analysis, | Matlab | Radiomics signature (Rad score) | R, nomogram |
| Zhong China, 2020 | DFS | - | Python DL | Radiomics signature (Rad score) | Matlab, R, nomogram, Kaplan Meier survival curves |
| Dong China, 2019 | FFS | ICC, univariate p_interaction_ value, backward stepwise selection | Matlab | multivariable Cox proportional hazard model | R, Kaplan Meier survival curves |
| Yang China, 2022 | Treatment Response | Univariate analysis, recursive feature addition | Python DL Pyradiomics | SVM | Python, R, ROC |
| Hu China,  2021 | Treatment Response | mRMR, LASSO | AI Kit software | Radiomics signature (Rad score) | SPSS, R, ROC, nomogram, DCA |
| Liao China, 2021 | Treatment Response | ICC, single-factor analysis, LASSO | Python Pyradiomics | Multivariate logistic regression, BPNN | Python, R, Matlab, ROC, nomogram, DCA |

AIC, Akaike information criterion; AI Kit software, Artificial Intelligence Kit software; DCA, decision curve analysis; DL, deep learning; ICC, intra-observer intraclass correlation coefficient; mRMR, minimum redundancy maximum relevance; LASSO, the least absolute shrinkage and selection operator method; RF, random forest; SVM, support vector machine.

**References**

S1. Lambin P, Leijenaar RTH, Deist TM, et al. Radiomics: the bridge between medical imaging and personalized medicine. Nat Rev Clin Oncol. 2017; 14(12):749-62.
